# USING RE-AIM TO GUIDE INTERVENTION DEVELOPMENT AND PILOT TESTING: CASE STUDY OF THE TLC INTERVENTION

**DOI:** 10.1093/geroni/igad104.0153

**Published:** 2023-12-21

**Authors:** Rebecca Utz, Amber Thompson, Cathi Sparks, Bob Wong, Eli Iacob, Alexandra Terril, Mike Caserta

**Affiliations:** University of Utah, Salt Lake City, Utah, United States; University of Utah, Salt Lake City, Utah, United States; University of Utah, Salt Lake City, Utah, United States; University of Utah, Salt Lake City, Utah, United States; University of Utah, Salt Lake City, Utah, United States; University of Utah, Salt Lake City, Utah, United States; University of Utah, Salt Lake City, Utah, United States

## Abstract

Research-based interventions are often not fully translated or implemented into health or community settings, even after assessing and establishing feasibility and efficacy during pilot tests and randomized trials. The Time for Living and Caring (TLC) study, an intervention designed to improve respite care for dementia caregivers funded by NIA R01-AG061946, employed the principles of the RE-AIM framework to guide stage-1 research activities related to intervention (re)development and pilot testing, with the hope that implementation could be achieved more quickly and efficiently. RE-AIM is a framework used to guide dissemination and implementation activities by evaluating an intervention in terms of its: Reach to target populations; Effectiveness or efficacy; Adoption by target staff, settings, systems and communities; Implementation consistency, costs and adaptions made during delivery; and Maintenance/sustainment of intervention effects in individuals and settings over time. This presentation will describe the types of data used to evaluate each component of RE-AIM during the study’s stage-1 activities. Data include feedback from a community advisory board (n=12), a randomized waitlist-control sample evaluating feasibility and initial efficacy of intervention of the TLC intervention (n=163), and feedback from stakeholders and providers who would be partners in the implementation of the TLC intervention (n=59). Based on stakeholder feedback, our findings support recommending adopting and designing interventions with an eye toward the principles of RE-AIM as best practices to increase the likelihood that interventions developed in the research context can be translated and implemented to those who will benefit from them.

